# Genetic and metabolic effects of ripening mutations and vine detachment on tomato fruit quality

**DOI:** 10.1111/pbi.13176

**Published:** 2019-06-11

**Authors:** Sonia Osorio, Raphael T. Carneiro, Anna Lytovchenko, Ryan McQuinn, Iben Sørensen, José G. Vallarino, James J. Giovannoni, Alisdair R. Fernie, Jocelyn K. C. Rose

**Affiliations:** ^1^ Max‐Planck‐Institut für Molekulare Pflanzenphysiologie Potsdam‐Golm Germany; ^2^ Department of Molecular Biology and Biochemistry Instituto de Hortofruticultura Subtropical y Mediterránea “La Mayora” University of Malaga‐Consejo Superior de Investigaciones Científicas Málaga Spain; ^3^ Plant Biology Section School of Integrative Plant Science Cornell University Ithaca NY USA; ^4^ Boyce Thompson Institute for Plant Research and USDA‐ARS Robert W. Holley Center Ithaca NY USA

**Keywords:** fruit quality, mutants, tomato ripening

## Abstract

Tomato (*Solanum lycopersicum*) fruit ripening is regulated co‐operatively by the action of ethylene and a hierarchy of transcription factors, including *RIPENING INHIBITOR* (*RIN*) and *NON‐RIPENING* (*NOR*). Mutations in these two genes have been adopted commercially to delay ripening, and accompanying textural deterioration, as a means to prolong shelf life. However, these mutations also affect desirable traits associated with colour and nutritional value, although the extent of this trade‐off has not been assessed in detail. Here, we evaluated changes in tomato fruit pericarp primary metabolite and carotenoid pigment profiles, as well as the dynamics of specific associated transcripts, in the *rin* and *nor* mutants during late development and postharvest storage, as well of those of the partially ripening *delayed fruit ripening* (*dfd*) tomato genotype. These profiles were compared with those of the wild‐type tomato cultivars Ailsa Craig (AC) and M82. We also evaluated the metabolic composition of M82 fruit ripened on or off the vine over a similar period. In general, the *dfd* mutation resulted in prolonged firmness and maintenance of quality traits without compromising key metabolites (sucrose, glucose/fructose and glucose) and sectors of intermediary metabolism, including tricarboxylic acid cycle intermediates. Our analysis also provided insights into the regulation of carotenoid formation and highlighted the importance of the polyamine, putrescine, in extending fruit shelf life. Finally, the metabolic composition analysis of M82 fruit ripened on or off the vine provided insights into the import into fruit of compounds, such as sucrose, during ripening.

## Introduction

When fleshy fruits ripen, they typically undergo changes in colour, flavour, aroma and texture, all of which promote their consumption in order to facilitate seed dispersal (Lorts *et al*., 2008). These processes are associated with a broad range of metabolic pathways, including those that lead to the accumulation of sugars, organic acids and secondary metabolites, as well as the production of volatile organic compounds (Causse *et al*., 2010; Klee and Giovannoni, 2011; Rambla *et al*., 2014; Seymour *et al*., 2011; Tieman *et al*., 2012; Tohge *et al*., 2014). These ripening associated pathways are fundamental determinants of nutritional and organoleptic value. The later stages of ripening are also generally accompanied by substantial changes in texture, resulting from cell wall and middle lamella disassembly, as well as transpirational water loss (Seymour *et al*., 2013a; Uluisik *et al*., 2016; Vicente *et al*., 2007; Wang *et al*., 2018). Ultimately, these processes can lead to ‘over‐ripening’ and a loss of palatability (Seymour *et al*., 2013a).

Understanding the mechanistic basis of ripening regulation and the transition to fruit spoilage has been the focus of a multi‐billion‐dollar industry, and two general commercial approaches are used to enhance the postharvest longevity of fruit. The first involves controlling ripening through early harvest, although after the fruit are harvested their high metabolic rates can lead to rapid senescence (Gapper *et al*., 2013). Controlled atmosphere storage can slow this process to varying degrees (Díaz de León‐Sánchez *et al*., 2009; Maul *et al*., 2000), but does not fully prevent spoilage. The second approach relies on varieties that exhibit late or partial ripening, many of which were historically identified through genetic screens and selective breeding (Giovannoni, 2007; Kovács *et al*., 2009; Raffo *et al*., 2012). Both strategies are widely used in the production of tomato (*Solanum lycopersicum*) fruit, one of the most important horticultural crops worldwide (http://faostat3.fao.org/home/E). Tomato fruit are defined as climacteric and require the gaseous hormone ethylene to ripen. Indeed, tomato has emerged as the pre‐eminent experimental model for studying fleshy fruit, including the developmental control of ripening and ethylene synthesis and perception (Carrari and Fernie, 2006; Gapper *et al*., 2013; Giovannoni, 2004; Giovannoni *et al*., 2017; Hyang *et al*., 2009; Osorio *et al*., 2011; Seymour *et al*., 2013b).

One of the advantages of tomato as a model to study postharvest metabolism is the availability of pleiotropic non‐ripening mutants, including the mutants, *non‐ripening* (*nor*) and *ripening inhibitor* (*rin*), as well as the more recently identified *delayed fruit ripening* (*dfd*) genotype, all of which are impaired in multiple ripening‐related processes (Giovannoni, 2004; Saladié *et al*., 2007; Vrebalov *et al*., 2002). Unlike *nor* and *rin* fruit, those of *dfd* exhibit partial ripening, based on a number of canonical ripening‐related traits, although whole‐fruit firmness is prolonged for many months after the onset of ripening (Saladié *et al*., 2007). More modest increases in shelf life and resistance to pathogens have resulted from modifying the expression of genes associated with specific metabolites. Examples include malate‐mediated modifications of cellular redox levels (Centeno *et al*., 2011) and select transcription factors that were engineered into tomato fruit to increase anthocyanin content (Butelli *et al*., 2008; Zhang *et al*., 2013). These unanticipated phenotypes further underline the complexity and poorly understood molecular bases of textural changes and fruit deterioration.

Nonetheless, germplasm and molecular genetic tools are now available to substantially extend tomato ‘shelf life’, when defined in terms of softening, water loss and resistance to postharvest infection, for up to several months. Indeed, the *nor* and *rin* mutations have already been introduced into numerous commercial cultivars (Agar *et al*., 1994; Gardner, 2006; Garg *et al*., 2008; Kitagawa *et al*., 2005). Alternatively, a similar outcome can be achieved through the widely adopted commercial strategy of harvesting preripe fruit and inducing ripening with postharvest ethylene treatments (Guillén *et al*., 2007; Hurr *et al*., 2005; Jeong *et al*., 2004). However, it is not known whether either of these approaches, which focus on maintaining textural quality, are desirable from the perspective of nutritional and/ or flavour attributes.

To elucidate the complex molecular processes that underlie tomato ripening, large‐scale studies at the levels of the transcriptome, proteome and metabolome have been developed (Mohorianu *et al*., 2011; Osorio *et al*., 2011; Pan *et al*., 2014; Rohrmann *et al*., 2011; Shinozaki *et al*., 2018; Zhong *et al*., 2013). However, to date, postharvest aspects of tomato fruit biology have not been targeted. In this study, we investigated the primary metabolome, pigmentation and ripening‐related gene expression of fruit from the *nor*,* rin* and *dfd* ripening impaired genotypes, which remain firm and show no loss of integrity for long periods. These were contrasted with fruit of the normally softening ‘Ailsa Craig’ (AC) and M82 wild‐type varieties, which exemplify fresh market salad and processing tomato cultivars, respectively. These cultivars are widely used as experimental models and a comparison of the two provided a view of inter‐cultivar variation. In addition, we studied the metabolic changes that occur when M82 fruit ripen on (naturally) or off (commercial practice) the vine. The combination of metabolite and targeted transcript analyses allowed us to assess whether a metabolic compromise was incurred, with respect to key nutritional and/or organoleptic properties, upon: (a) prolonging visual/ textural quality traits and increasing storage potential through the use of mutations in genes that regulate ripening; or (b) by the practice of preripe fruit harvest.

## Results

### Postharvest characteristics of the ripening mutants

**Figure 1:**
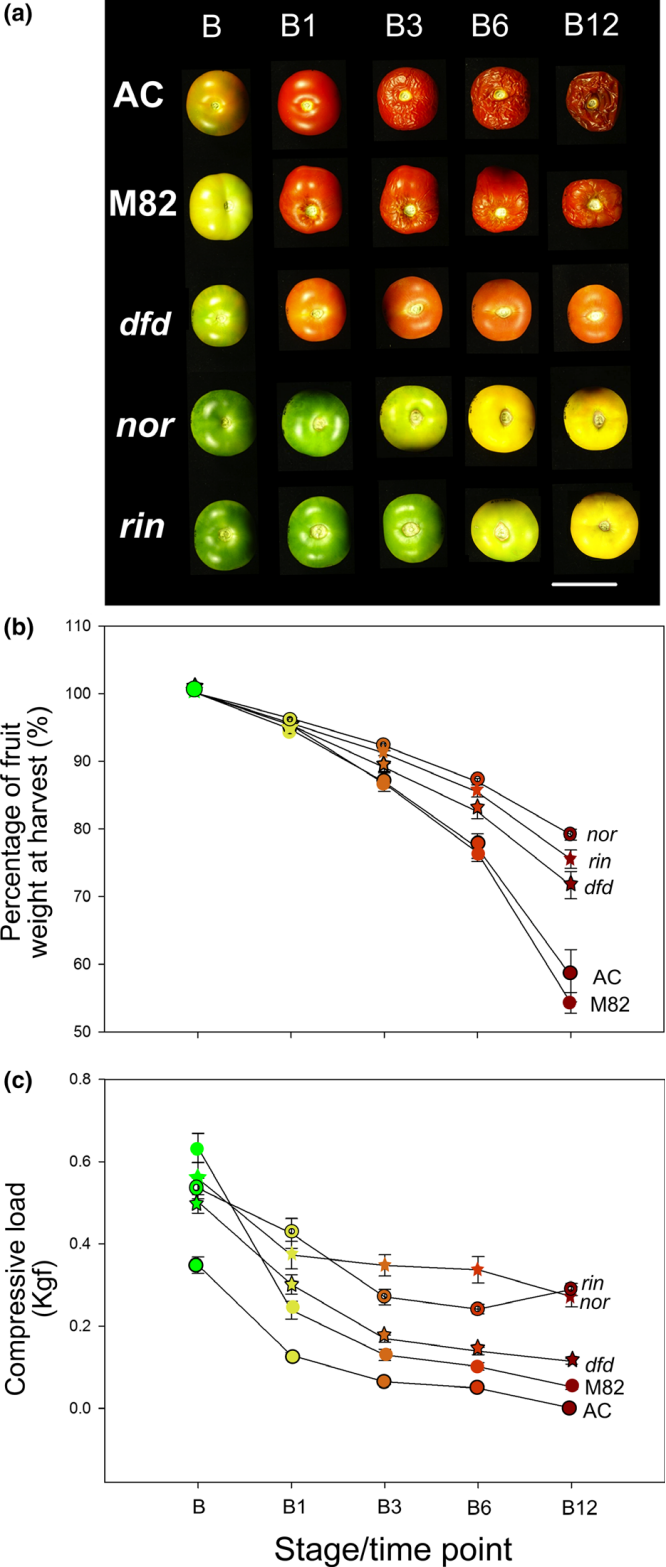
A comparison of AC, M82, *nor, rin* and *dfd* fruit ripening. (a) Ripening series of AC, M82, *dfd*,* nor* and *rin* at different time points (green, G; breaker, B; breaker + 1 week, B1; breaker + 2 weeks, B2; breaker + 3 weeks, B3; breaker + 4 weeks, B4; breaker + 6 weeks, B6; breaker + 12 weeks, B12). Scale bar = 5 cm. (b) Weight of fruit harvested at the B stage and stored for up to 12 weeks. (c) Firmness of intact fruit over the 12 weeks. Values correspond to the mean ± SE, *n *= 20.

The three mutant tomato genotypes being studied, *nor*,* rin* and *dfd*, are known for their impaired fruit ripening phenotypes, and as a first step we performed a comparative analysis, focusing on commercially important ripening‐related traits: external colour, postharvest desiccation and fruit firmness. Fruit from *dfd, nor* and *rin*, as well as those from the normally softening wild‐type (WT) cultivars AC and M82, were harvested at the breaker (B) stage, or equivalent based on days after pollination (DAP), and were stored at room temperature for up to twelve weeks. Over this time course, the WT fruit ripened fully and showed clear over‐ripe phenotypes (e.g. extensive tissue collapse and desiccation) by 12 weeks after the breaker stage (B12). In contrast, the mutant fruits exhibited varying degrees of delayed postharvest ripening phenotypes, including major differences in colour changes (Figure 1a), reduction in weight, which was taken as an indication of water loss (Figure 1b), and firmness, defined here as resistance of intact fruit to compression (Figure 1c). Fruit of *nor* and *rin* displayed the most substantial differences in all these parameters, compared to the AC and M82 genotypes, while *dfd* fruit showed an intermediate phenotype. The substantial water loss from AC and M82 fruit between B3 and B12 coincided with increasing external wrinkling and tissue collapse (Figure 1c), establishing the period after B3 as the time during which palatability was lost. We also noted that AC fruit showed the lowest resistance to compression at the B stage, while those of the M82 were the firmest, although both softened to similar final values of compression load.

### Postharvest‐related changes in metabolite levels in the mutant fruits

**Figure 2:**
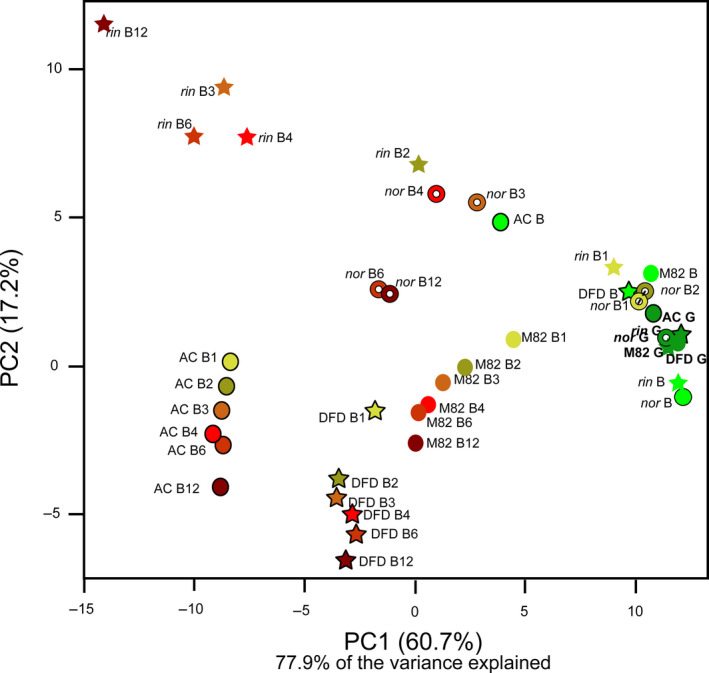
Principal component analysis (PCA) of primary and carotenoid metabolites of AC, M82, *dfd*,* nor* and *rin* tomato fruit across ripening and postharvest stages (green, G; breaker, B; breaker + 1 week, B1; breaker + 2 weeks, B2; breaker + 3 weeks, B3; breaker + 4 weeks, B4; breaker + 6 weeks, B6; breaker + 12 weeks, B12). Data were collected from two different platforms as described in Experimental Procedures.

One of the main aims of this study was to investigate the consequences of extending tomato fruit shelf life on the primary and pigment metabolomes. We analysed primary metabolites in the same fruit samples using an established gas chromatography–mass spectrometry (GC‐MS) method (Fernie *et al*., 2004). The combined data sets were examined by principal component analysis (PCA) (Figure 2), which revealed two distinct patterns. At earlier ripening stages, from the green stage (G) to B1, the genotypes and stages were essentially indistinguishable; however, at later postharvest stages (from B2 to B12), clear differences were evident between genotypes, with PC1 and PC2 explaining >77% of the variance. For example, there was a sharp time‐dependent trend towards negative values on both axes for the normally ripening M82 and AC varieties and the *dfd* mutant, whereas values for *nor* and *rin* fruit were more clustered and displayed a delayed decrease (Figure 2). As with the water loss and firmness traits, the *dfd* genotype was intermediate between the grouped *nor* and *rin* mutants and the WT genotype. These findings are largely in accordance with previous metabolic studies of both *nor* and *rin*, which highlighted only relatively minor changes in metabolite content in the mutant fruit prior to harvest (Osorio *et al*., 2011). In addition, loading plots indicated that the separation by PC1 is largely based on changes in a few tricarboxylic acid (TCA) cycle intermediates (succinate and fumarate), γ‐carotene, *cis*‐lycopene, several amino acids (methionine (Met), alanine (Ala), tyrosine (Tyr), aspartate (Asp), and glutamate (Glu)) and metabolites related to ascorbic acid (vitamin C) metabolism (galacturonic, threonic and dehydroascorbic acids). On the other hand, PC2, which resolved the *rin* and *nor* mutants from AC, M82 and *dfd,* was related mainly to differences in phytofluene, malate and the two main sugars, fructose (Fru) and glucose (Glc) (Table S1).

**Figure 3:**
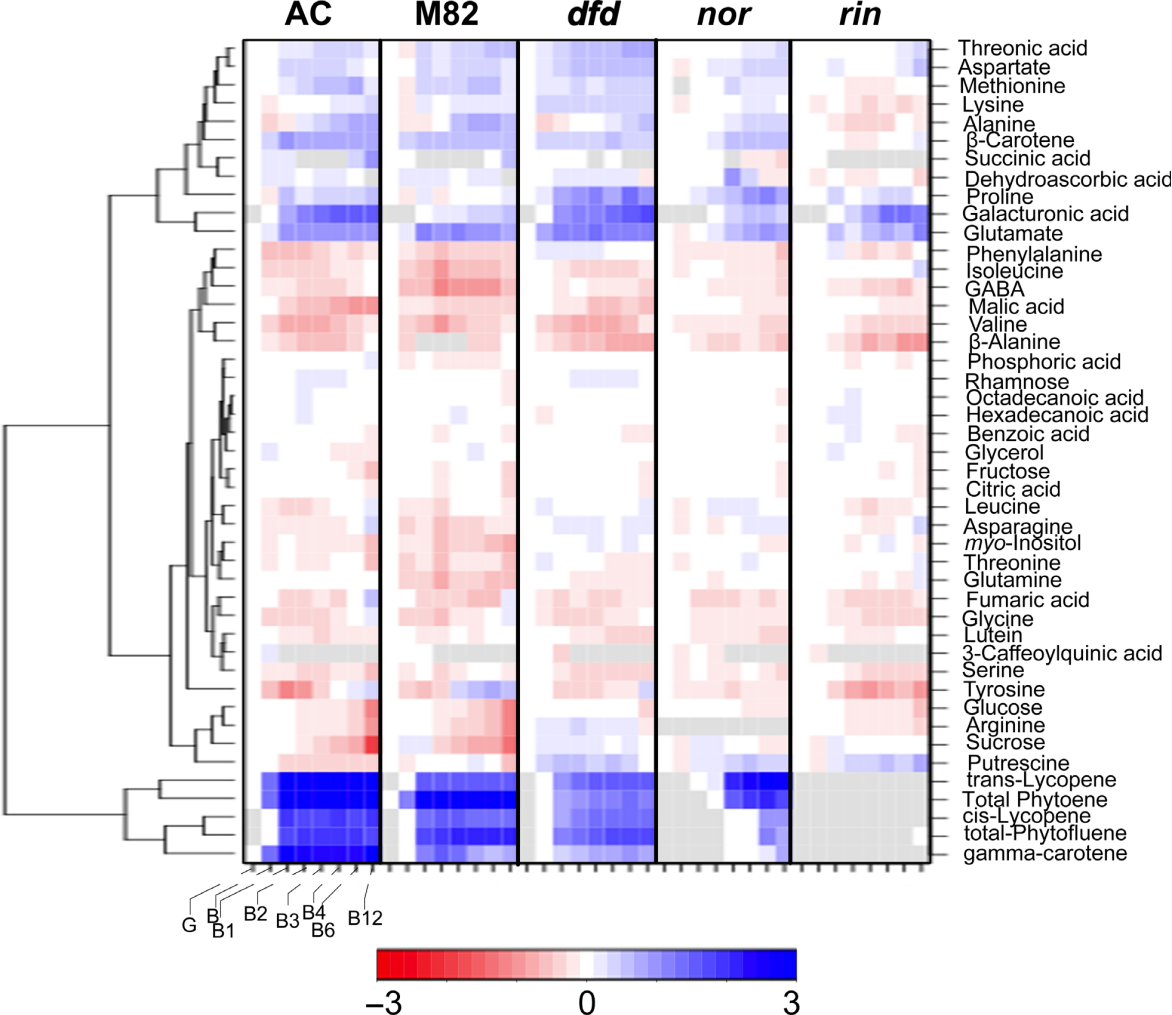
Hierarchical cluster analysis of the changes in primary and carotenoid metabolism in AC, M82, *dfd*,* nor* and *rin* during tomato ripening and postharvest stages (green, G; breaker, B; breaker + 1 week, B1; breaker + 2 weeks, B2; breaker + 3 weeks, B3; breaker + 4 weeks, B4; breaker + 6 weeks, B6; breaker + 12 weeks, B12). Data are normalized to the mean response calculated for the green stage for each genotype (*n *= 6). The scale is logarithmic. Values are displayed in false‐colour code.

The most obvious visual characteristic of fruit ripening is colour change (Figure 1a), involving the transition from chloroplasts in green fruit to chromoplasts in ripe fruit. This is accompanied by qualitative and quantitative changes in the profile of carotenoids, and so in parallel with the primary metabolite profiling, we examined the differential accumulation of the carotenoids phytoene, phytofluene, *trans*‐lycopene, *cis*‐lycopene, ß‐carotene, γ‐carotene and lutein at eight stages of ripening and postharvest storage, from G to B12. In chloroplast containing tissues, xanthophylls (zeaxanthin and lutein) predominate, reflecting their association with photosynthetic function. Accordingly, we observed a decrease in lutein in all genotypes during ripening and in the postharvest stages (Figure 3; Table S2). In chromoplast‐containing tissues, however, acyclic carotenoids (phytofluene, all carotenoid isoforms and lycopene) accumulated. Interestingly, the comparison of AC, M82 and *dfd* mutant revealed a similar pattern, although the abundance of these carotenoids was reduced in *dfd* (Figure 3; Table S2). This result is in agreement with the lighter red colour observed in *dfd* fruit (Figure 1a). The levels of this group of carotenoids were also higher in the *nor* mutant, but showed a delay in their increase until later postharvest stages (from B4 to B12; Figure 3; Table S2). Of the major carotenoids, only ß‐carotene was detected in the *rin* mutant. However, while this pigment increased in abundance during normal ripening and postharvest in AC, M82, *dfd*, and *nor*, it decreased during postharvest in *rin* (Figure 3; Table S2).

We observed canonical changes in primary metabolites during ripening (Carrari *et al*., 2006). Of the compounds analysed, more than 85% changed significantly during ripening and postharvest (*P *< 0.05) (Figure 3). During normal postharvest (i.e. in the AC and M82 genotypes), glucose (Glc) and sucrose (Suc) levels decreased, as did the main intermediates of the TCA cycle although citric acid abundance was only lower at later stages of postharvest. Levels of the cell wall associated sugars galacturonic acid, and rhamnose in the case of AC and *dfd,* increased in content during postharvest, consistent with their release during the degradation of cell wall pectin that occurs in late ripening. We noted that the magnitude of these increases was considerably greater in AC than in M82. Levels of other organic acids, such as threonic and dehydroascorbic acid, both of which are involved in vitamin C biosynthesis, also generally increased during postharvest.

Although the levels of the amino acids aspartate (Asp), methionine (Met), lysine (Lys), alanien (Ala), proline (Pro) and glutamate (Glu) were higher in both WT genotypes, those of most other amino acids were lower, while glutamine (Gln) levels were unaltered during postharvest in AC (Figure 3; Table S3). In contrast, serine, fumarate and 3‐caffeoylquinic acid followed a similar pattern of decreasing abundance during ripening in both AC and M82 genotypes, while asparagine (Asn), benzoic and phosphoric acid, glycerol, and hexadecanoic and octadecanoic acid maintained consistent levels at the G stage in both genotypes and displayed similar patterns of change during ripening. Despite similar general temporal changes in metabolite levels in AC and M82, several differences were apparent. Namely, we observed a large decrease in putrescine during ripening and storage in AC that did not occur in M82, whereas Glu levels decreased in M82, but not in AC. In contrast, there was a large increase in Tyr at the later stages of ripening in M82, but not in AC.

A comparison of the metabolites present in fruit of the three mutants during postharvest with those in the AC and M82 genotypes indicated that approximately 60% were altered in both the *nor* and *rin* mutants. However, the metabolite changes in *dfd* during postharvest were very similar to those of the WT genotypes (Figure 3; Table S3). Only a few metabolites in *dfd* showed the same differences during postharvest storage as in the *nor* and *rin* mutants. These included the amino acid γ‐aminobutyric acid (GABA), the major sugars glucose (Glc) and sucrose (Suc), and the polyol *myo*‐inositol (Figure 3; Table S3). However, in several cases, changes in metabolite levels in *dfd* were intermediate between those seen in the control genotypes and in *nor* and *rin*. These included decreases in β‐alanine (β‐Ala), threonine (Thr) and malate, and increases in methionine (Met), which were delayed or abolished in *nor* and *rin*, but only slightly delayed in *dfd*. That said, the changes in the levels of five amino acids did not follow this pattern. The typical decreases in leucine (Leu) and isoleucine (Iso) were absent in *dfd* and *nor* but merely delayed in *rin*, whereas the normal decrease in phenylalanine (Phe) levels was absent in *dfd* but merely delayed in *nor* and *rin*. Furthermore, Lys levels were twofold higher throughout ripening and storage, but only somewhat lower in *rin*, while major increases in the levels of Pro were observed in *dfd* and *nor,* but not in *rin*. However, most of the other changes in *nor* and *rin* were similar to those associated with the normal postharvest changes observed in AC and M82, but were merely delayed. For example, levels of glucose (Glc), sucrose (Suc) and malic, threonic and dehydroascorbic acids were unchanged or only changed slightly, during postharvest in both *nor* and *rin* (Figure 3; Table S3). Levels of galacturonic acid increased in *nor* and *rin* during later stages of postharvest, consistent with a delay in cell wall depolymerization (Zheng *et al*., 1994). In keeping with this, the increase in rhamnose, observed in the controls and in *dfd,* was abolished in *rin* and *nor*, possibly reflecting reduced pectin degradation. Similarly, other metabolites whose levels changed during postharvest in the WT genotypes, such as alanine (Ala), isoleucine (Ile), aspartate (Asp), methionine (Met) and lysine (Lys), showed no changes in abundance in the mutants, or changes that were delayed to a later stage of postharvest (Figure 3; Table S3).

### Changes in transcript levels associated with carotenoid metabolism and other ripening‐related processes

**Figure 4:**
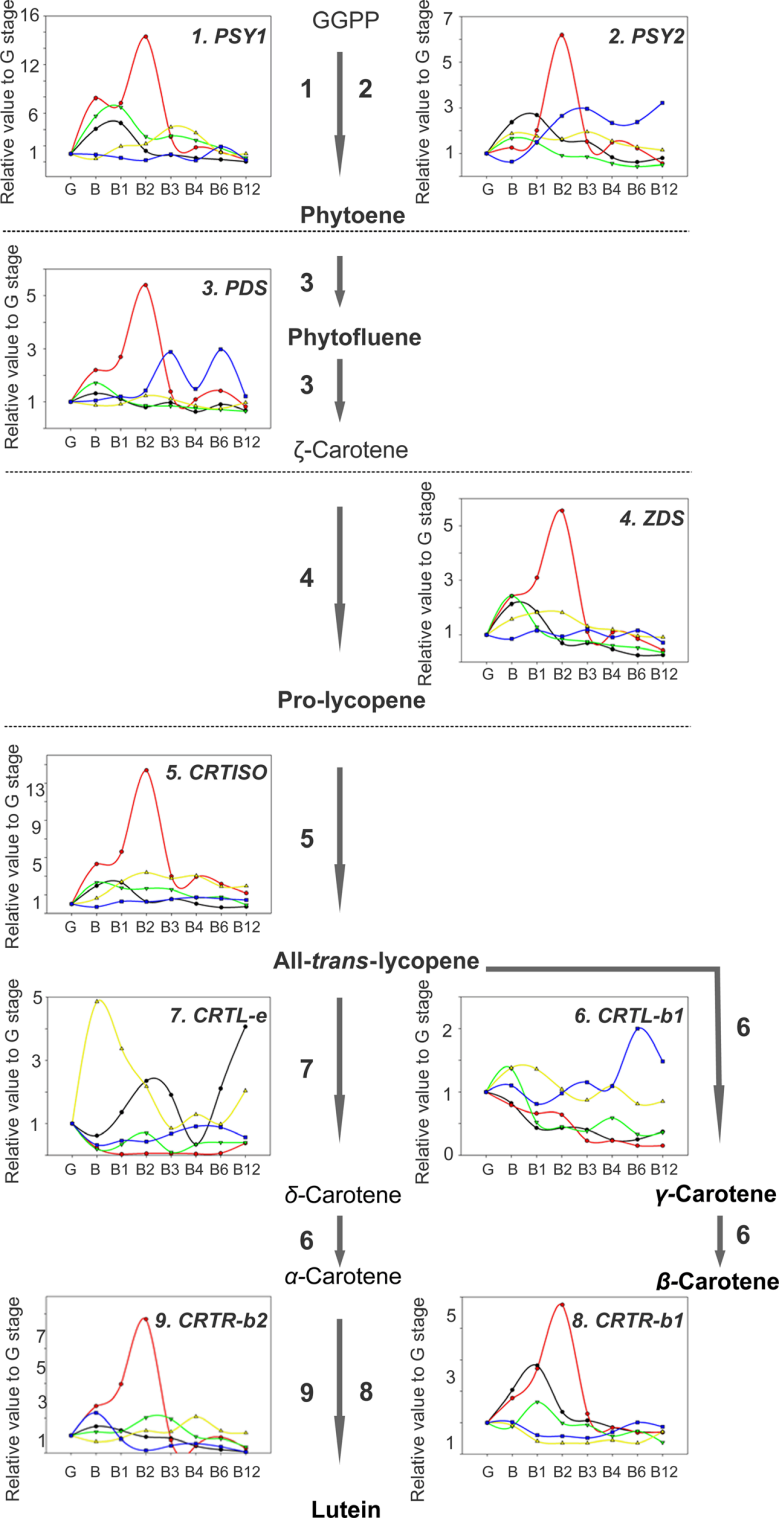
Expression analysis of genes related to carotenoid biosynthesis in the AC (black line), M82 (red line), *dfd* (green line), *nor* (blue line) and *rin* (yellow line) genotypes during tomato ripening and postharvest stages (green, G; breaker, B; breaker + 1 week, B1; breaker + 2 weeks, B2; breaker + 3 weeks, B3; breaker + 4 weeks, B4; breaker + 6 weeks, B6; breaker + 12 weeks, B12) by quantitative real‐time PCR (*n *= 6). Changes in gene expression levels were determined with gene‐specific primers for (1) *phytoene synthase 1, PSY1*, (2) *phytoene synthase 2, PSY2*, (3) *phytoene desaturase*,* PDS*, (4) zeta‐carotene desaturase, *ZDS*, (5) *carotene isomerase*,* CRTISO*, (6) *lycopene ß‐cyclase*,* CRTL‐b1*, (7) and *ε‐ring hydrolase*,* CRTL*. Geranylgeranyl diphosphate, GGPP. The measured carotenoid levels are indicated in bold font (see Figure 3).

We next measured the expression levels of several known carotenoid‐related genes by quantitative real‐time PCR (qRT‐PCR) during postharvest. These included genes encoding the core enzymes phytoene desaturase (*PDS*), zeta‐carotene desaturase (*ZDS*), phytoene synthase (*PSY1* and *PSY2*), ß‐carotene hydrolase (*CRTR‐b1* and *CRTR‐b2*), ε‐ring hydrolase (*CRTR‐e*), lycopene ß‐cyclase (*CRTL‐b1* and *CRTL‐e*) and carotene isomerase (*CRTISO*). There was a clear difference in the dynamics of gene expression in the mutants compared with AC and M82, most prominently in *rin* and *nor*. However, the trends did not always mirror those observed at the primary metabolite or carotenoid levels (Figure 4). We noted that the expression of *CRTL‐e,* which is involved in lutein biosynthesis, was highly induced at earlier ripening stages in the *rin* mutant, which is in agreement with the higher lutein levels in earlier ripening stages in this mutant (Figure 3). In summary, with the exception of *CRTL‐e*, the normal postharvest increase in gene expression was either delayed or absent (in the *nor* and *rin* mutants), or of lower magnitude (in *dfd;* Figure 4).

**Figure 5:**
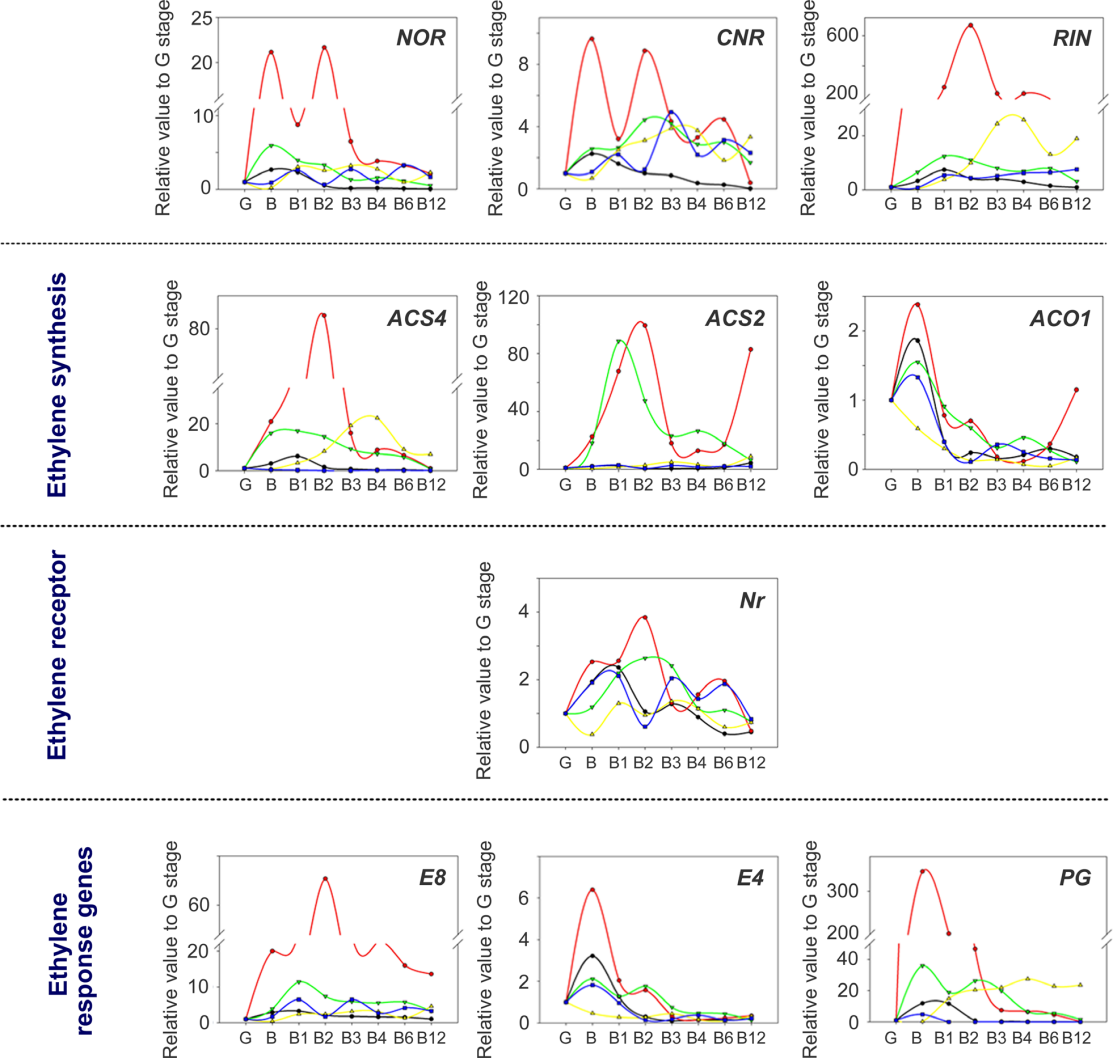
Changes in ethylene‐related gene expression levels in the AC (black line), M82 (red line), *dfd* (green line), *nor* (blue line) and *rin* (yellow line) genotypes during tomato ripening and postharvest stages (green, G; breaker, B; breaker + 1 week, B1; breaker + 2 weeks, B2; breaker + 3 weeks, B3; breaker + 4 weeks, B4; breaker + 6 weeks, B6; breaker + 12 weeks, B12), measured by quantitative real‐time PCR (*n *= 6).

We also investigated the expression of a set of ripening‐related genes associated with ethylene biosynthesis (*ACS2, ACS4, ACO1*; Nakatsuka *et al*., 1998); ethylene responses (*E4, E8*, Lincoln and Fischer, 1988); the transcription factors necessary for ethylene synthesis, *NOR, RIN* and *CNR* (Manning *et al*., 2006; Vrebalov *et al*., 2002); the ethylene receptor, *NEVER RIPE*, (*Nr*); and the cell wall degradation associated polygalacturonase (*PG*; Dellapenna *et al*., 1986; Giovannoni *et al*., 1989). The patterns of gene expression of ethylene‐related genes showed major differences between the genotypes (Figure 5). In M82, the changes in relative gene expression were much greater than for the other genotypes, including AC (Figure 5). The expression profiles of *NOR* and *CNR* were similar in all genotypes, and while levels of both genes were higher at the onset of ripening (stage B), peak expression was delayed in the *nor* and *rin* mutants, and of a lower magnitude in *dfd* (Figure 5). Similarly, the transcript levels of *RIN* peaked at the onset of ripening in all genotypes, with the exception of the *rin* mutant, where the peak expression was shifted to later stages (Figure 5). *ACS4* and *ACO1* were induced in M82, AC and *dfd* ripening fruit, as reported previously for normal ripening (Nakatsuka *et al*., 1998), whereas *ACS2* was only induced in M82 and *dfd* (Figure 5). The accumulation of *ACS4* mRNA was delayed in *rin* but completely absent in *nor*, whereas the expression of *ACO1* declined most rapidly in *rin* (Figure 5). Levels of expression of *E4*,* E8* and *PG* showed a slight to moderate induction in AC, M82 and the *dfd* mutant (Figure 5). In agreement with the suppressed ripening of *rin* (Figure 1a), we observed unchanged transcript levels of *E8* and *E4* during ripening and postharvest stages, as well as a shift in the accumulation of *PG* transcripts to later stages (Figure 5). In contrast, the pattern of expression of these genes in *nor* was similar to that of AC, M82 and *dfd*, although changes were lower in magnitude (Figure 5).

### Comparative metabolomic analysis of on‐vine or off‐vine ripened tomato fruit

In addition to utilizing genetic variation to prolong shelf life, a key strategy for most large‐scale commercial tomato production worldwide involves harvesting fruit at a preripe stage and then triggering off‐vine climacteric ripening by exposure to ethylene (Kader, 1986). This has the clear advantage of allowing the maintenance of firmness during shipping and storage, thereby reducing spoilage. However, detaching fruit also precludes further delivery of nutritionally valuable compounds from the parent plant. While this fact is widely acknowledged, to date, there has been no large‐scale study of the metabolic consequences of off‐vine ripening.

**Figure 6:**
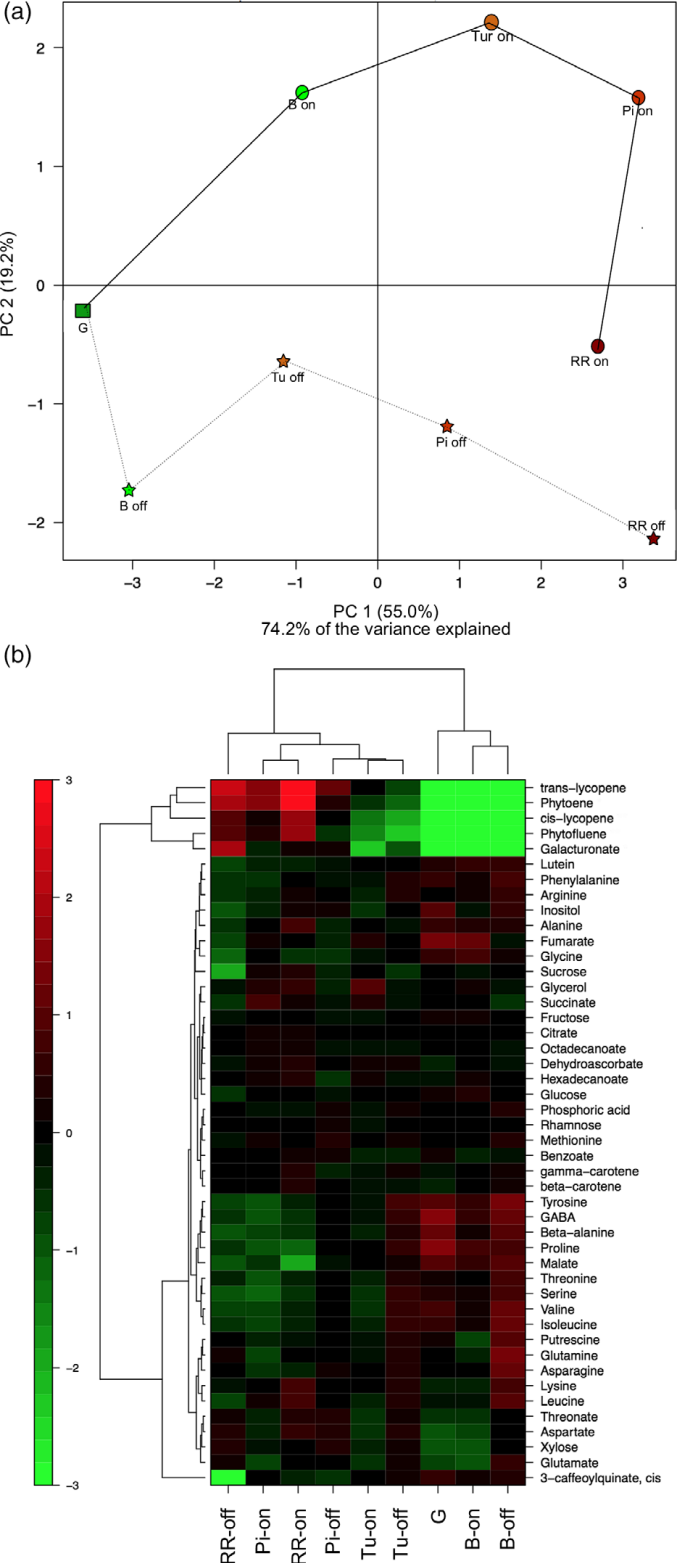
Overview of the changes in primary and carotenoid metabolism in on‐vine or off‐vine ripened M82 fruits. The analysed ripening stages were green (G), breaker (B), turning (Tu), pink (Pi) and red (RR). (a) Principal component analysis (PCA). (b) Heatmap representation. Data are normalized to the mean response calculated for the green stage (*n *= 6). The scale is logarithmic. Values are displayed in false‐colour code.

We used GC‐MS to quantify 46 metabolites from tomato fruit at five developmental stages based on external colour (Lashbrook *et al*.,^1994^): G, B, Turning (Tu), Pink (Pi) and Red Ripe (RR) fruit of the M82 cultivar were ripened on the vine, or harvested at the G stage and allowed to ripen postharvest. The full data set from this study was then analysed by PCA (Figure 6a). The first principal component (PC1), which explains 55.0% of the total variance, separated all the ripening stages. In contrast, the second principal component (PC2), which explains 19.2% of total variance, clearly separated samples that were ripened on and off the vine. We next performed a correlation analysis across the entire metabolite data set. The analysis of all ripening stages of tomato fruit ripened on the vine revealed 109 significant correlations (*P *< 0.05), of which 77 were positive and 32 negative (Figure S1). When the same analysis was performed using data obtained from fruit ripened off the vine, a total of 160 correlations were observed of which 153 were positive and only seven negative (Figure S1). The amino acids ß‐Ala, Val, Tyr, Thr, Ser, Phe and Ile displayed strong correlations with one another under both conditions.

We then performed a hierarchical cluster analysis across all ripening stages of fruit ripened on and off the vine (Figure 6b), which revealed two main clusters. One contained the more immature stages (stages G, and B), of both ripening types. In addition, Tu on or off the vine, and Pi off the vine stages clustered together, indicating similar metabolite profiles. However, fruit at the RR stage ripened on or off the vine clustered independently of one another, indicating that their metabolic composition had diverged by this developmental stage (Figure 6b).

The levels of the predominant carotenoids increased similarly during ripening, irrespective of ripening type (Figure 6b). Galacturonic acid also showed a large increase in abundance during ripening, but was higher in RR fruit ripened off the vine. Of the non‐volatile compounds, key contributors to taste in tomato fruit are the major sugars (glucose, fructose and sucrose) and organic acids (malic and citric acid). Notably, when comparing the RR stage of the two ripening types, we observed that the levels of the three major sugars were lower in off‐vine ripened fruit while the level of malic acid was higher (Figure 6b).

At a quantitative level, the most marked changes were lower levels of alanine (Ala), arginine (Arg), leucine (Leu) and carotenoids and much lower levels of sucrose (Suc) and lysine (Lys) in the off‐vine ripened fruit, together with substantially higher levels of putrescine. That these metabolites change is of interest since, as we discuss below, the levels of several of them have been reported to correlate with shelf life in previous studies (Dibble *et al*., 1988; Raffo *et al*., 2012; Rastogi and Davies, 1991). Some other amino acids were differentially accumulated in the ripe fruit, with proline (Pro) and asparagine (Asn) being higher and phenylalanine (Phe), tyrosine (Tyr), β‐alanine (β‐Ala), serine (Ser), valine (Val), leucine (Leu) and aspartate (Asp) being lower in fruit ripened off the vine.

## Discussion

In this study, we utilized a metabolomics approach and targeted transcriptional analysis to elucidate the potential effects on tomato fruit quality of delaying ripening and suppressing over‐ripening through the use of ripening mutations, and to contrast off‐vine and on‐vine ripening.

### Different regulation of colour development and softening

Characteristic changes in carotenoid accumulation were observed in AC and M82 during ripening and postharvest storage (Figures 1a and 3). In contrast, neither *rin* nor *nor* exhibited large increases in pigmentation at the B stage, and any change was either delayed to later postharvest stages (*nor*) or abolished (*rin*; Figures 1a and 3). Previous determinations of flux control coefficients indicated that phytoene synthase, *SlPSY1*, exerts considerable control of the flux of carbon into carotenoids (Bramley *et al*., 1992; Fraser *et al*., 2002). We observed an increase in *PSY1* expression during the earlier ripening stages in AC, M82 and the *dfd* mutant (from B to B2 stages; Figure 5), whereas this increase in gene expression occurred later in postharvest in *rin* and *nor* (from B3 to B6 stages; Figure 5). This differential *PSY1* expression may account, at least in part, for the increase of carotenoids in the WT varieties and *dfd*. This is consistent with results of a previous study (Martel *et al*., 2011) in which RIN was demonstrated to regulate carotenoid accumulation by interacting with the *PSY1* promoter. In contrast, *PSY2* has been shown to not influence the flux to carotenoids in ripening fruit (Bartley and Scholnik, 1993; Fraser *et al*., 1999). In agreement with this, we did not observe a signature *PSY2* expression pattern for normal or delayed ripening. It has been established that at the same time that *PSY* and *PDS* transcript levels increase, at the B stage of ripening, those of the lycopene cyclases, *CRTL‐e* and *CRTL‐b1* disappear (Pecker *et al*., 1996; Ronen *et al*., 1999) in response to the ethylene produced by the ripening fruit (Alba *et al*., 2005). The hypothesis that this differential gene expression underlies the accumulation of lycopene and β‐carotene is supported by our observation of the accumulation of both metabolites in the fruit of AC, M82 and *dfd*, as well as in *nor*, all of which additionally accumulate *PSY1* and *PDS* transcripts (Figures 3 and 5). These results support the proposal that the formation of lycopene and β‐carotene from phytoene is under tight transcriptional regulation (Corona *et al*., 1996; Hirschberg, 2001; Mann *et al*., 2000). Additionally, we observed a large increase in *CRTL‐e* expression at the B equivalent stage of the *rin* mutant, which can explain the increase in lutein levels during ripening and postharvest storage (Figures 3 and 5).

Another prominent ripening‐related change in tomato fruit is the progressive loss of firmness, a phenomenon that is limited in *rin* and *nor* fruit (Osorio *et al*., 2011). The firmness and weight loss data in this study corroborate previous observations. The *dfd* mutant displayed normal increases in carotenoid content (Figures 1a and 3) and an intermediate behaviour in terms of firmness and weight loss parameters between the normal softening varieties (AC and M82) and the two long shelf life mutants (*rin* and *nor;* Figure 1). This confirmed the previous observation that key ripening‐related traits in *dfd* are largely uncoupled from loss of intact fruit firmness (Saladié *et al*., 2007).

### Differential regulation of ethylene and polyamine biosynthesis in *rin, nor* and *dfd*

In agreement with previous studies (Osorio *et al*., 2011; Vrebalov *et al*., 2002; Wilkinson *et al*., 1995), we observed that ethylene‐related genes were differentially expressed in *rin* and *nor* compared to the WT cultivars and *dfd* (Figure 5). We observed that during ripening and postharvest, expression of the ethylene synthesis‐related genes, *ACS4*,* ACS2* and *ACO1,* was upregulated in B stage fruit. Interestingly, these genes either displayed no expression or delayed upregulation in *nor* and *rin*, which is consistent with the absence of climacteric ethylene production in both mutants (Giovannoni, 2007). However, the expression of genes in the downstream ethylene signalling cascade was apparently unaltered during ripening and postharvest across the genotypes (Figure 5). Analysis of the *Never‐ripe* (*Nr*) mutant revealed that carotenoid formation is dependent on ethylene perception (Alba *et al*., 2005), and our data suggest that this is also the case in the *dfd* mutant, as well as in WT cultivars. In contrast, carotenoid biosynthesis in the *nor* mutant was found to be independent of ethylene synthesis (Figures 3 and 5) since carotenoids accumulated in the apparent absence of ethylene production in later postharvest stages in this mutant, whereas carotenoid accumulation was absent in *rin* (Figure 5).

Ethylene is synthesized via the Yang cycle from S‐adenosyl methionine (SAM), which is also precursor for the formation of polyamines, such as putrescine (Mattoo and Handa, 2008; Mattoo *et al*., 2006). Ethylene and polyamines have been reported to possess opposing biological roles: ethylene promotes senescence, whereas polyamines are known to suppress it, by slowing down membrane deterioration and loss of chlorophyll and enhancing protease and RNAse activities (Sauter *et al*., 2013). Our metabolite data suggest that polyamine biosynthesis may dominate the flux through SAM in ripening impaired mutants. During normal tomato ripening, Asp and Met levels increased, while the abundance of putrescine, one of the three major polyamines, decreased (Figure 3). In contrast, the three long shelf life mutants displayed an increase in putrescine levels during ripening and postharvest, as previously described for another long shelf life tomato mutant, *alcobaça*, and a long shelf life cultivar, Liberty (Dibble *et al*., 1988; Saftner and Baldi, 1990). During normal tomato postharvest storage, ACC continues to be produced, but is not converted into ethylene, but is rather conjugated to its inactive storage form, malonyl‐ACC (Van de Poel *et al*., 2012). We hypothesize that this is the case in AC and M82 during postharvest, since we observed an increase in aspartate (Asp) and methionine (Met) levels, together with lower *ACO* expression. However, in *dfd,* although Asp and Met levels changed, as in the normally softening tomato cultivars, putrescine levels increased during ripening and postharvest, as in *rin* and *nor* (Figure 3). This hypothesis is strongly supported by the higher expression of SAM decarboxylase and spermidine synthase genes in the three ripening mutants compared with M82 at the B stage (Figure S2). These results indicate that an altered co‐ordination between the Yang cycle, ethylene biosynthesis and polyamine biosynthesis occurs in the mutants. Given the emerging role of polyamines as interactors with secondary messengers and mediators within several signalling pathways (Mattoo and Handa, 2008; Mattoo *et al*., 2006), we suggest that changes in alanine (Ala), which is a breakdown product of spermidine, and arginine (Arg), which is a precursor of polyamine biosynthesis, also reflect metabolic shifts at this important branch point. Indeed, it has been shown that labelled putrescine and spermidine are metabolized via GABA to the TCA cycle and ultimately to other amino acids and sugars (Rastogi and Davies, 1991). The data presented here contribute to the growing body of evidence correlating polyamine levels with extended shelf life (Karlova *et al*., 2011; Nambeesan *et al*., 2010; Saftner and Baldi, 1990). However, the complexity of the interaction between ethylene and polyamine mediated effects is illustrated by the fact that some of the phenotypes of ethylene suppressed tomatoes can be offset by introgression of the higher polyamine trait (Sobolev *et al*., 2014). Moreover, overexpression of SAM decarboxylase in tomato was reported to prevent the ripening associated depletion of polyamine levels without altering the levels of ethylene (Lasanajak *et al*., 2014). A better understanding of the interaction between ethylene and polyamines may help develop strategies to prolong shelf life, while retaining desirable ripening‐related traits.

### The *rin* and *nor* mutants display marked postharvest metabolic shifts in comparison with *dfd*

The levels of sucrose, glucose and fructose, the three major sugars in tomato fruit, decreased in the WT cultivars during postharvest storage, as is typical (see for example Cheema *et al*., 2014). In *rin* and *nor* fruit, however, we observed largely unaltered levels of these metabolites during postharvest, although their levels increased during postharvest storage in *dfd* (Figure 3). Moreover, a strong negative correlation was observed between the levels of Asp and its precursor malic acid, which decreased during ripening and postharvest in all genotypes, although this decrease was delayed in *rin* and *nor* (Figure 3). This finding is consistent with previous studies indicating that malate and redox status influence firmness during storage (Centeno *et al*., 2011; Zhang *et al*., 2013, 2015). In contrast, levels of citric acid were invariant, irrespective of developmental stage or genotype. These six metabolites are key to providing a foundation for desirable flavour (Baldwin *et al*., 2008).

Levels of the nutritionally valued compound, ascorbic acid (vitamin C), have been shown to decrease in tomato during postharvest storage (Cheema *et al*., 2014). In the current study, ascorbic acid was not detected, due to the nature of the extraction buffer used. However, we observed a high increase in levels of its oxidized form, dehydroascorbic acid (DHA) during ripening and postharvest storage of *dfd* and the normally softening tomato varieties, while DHA levels did not change, or slightly decreased, in *rin* and *nor* during postharvest. Moreover, we observed the same trends in threonic acid (a catabolic product of ascorbic acid) during ripening and postharvest in all genotypes. These data, together with those related to polyamines described above, suggest higher redox and carbon recycling activities in the normal softening tomato varieties and *dfd* in comparison with *nor* and *rin*.

Our results reveal that that the *dfd* fruit have a prolonged shelf life, compared with AC and M82, and are likely to have a more attractive taste profile and other nutritionally valuable attributes, than *rin* and *nor*.

### Effects of ripening on and off the vine on metabolite composition

We wanted to assess the metabolic consequences of detaching tomato fruit and ripening them off the vine and thereby infer the extent to which materials are imported from the plant to synthesize or maintain their metabolic composition. Tomato fruit were harvested at the G stage and left to ripen at room temperature, and then we periodically collected samples for metabolomic analysis and compared them with samples from the same ripening stages of fruit ripened on the vine. Our data reveal that on‐vine and off‐vine ripened tomatoes were comparable at earlier ripening stages; however, notable differences were observed at the RR stage, consistent with a study of fewer compounds using nuclear magnetic resonance (NMR) analysis (Sorrequita *et al*., 2013).

As a sink organ, the tomato fruit is supplied with carbon in the form of sucrose from photosynthetic tissue, which can be used for the biosynthesis of primary metabolites (Ho, 1984). Previous studies suggested that sucrose import ceases during tomato fruit ripening due to the formation of an abscission layer between the calyx and fruit (Balibrea *et al*., 2006; McCollum and Skok, 1960). However, we observed that sucrose and glucose levels in off‐vine ripened red fruit were fivefold lower than in on‐vine ripened fruit, suggesting that considerable sucrose delivery occurs beyond the time point at which the abscission zone forms. Consistent with this hypothesis, we observed that the elevated degree of metabolite‐to‐metabolite correlation in off‐vine ripened fruit was largely driven by amino acid‐to‐amino acid correlations, which may reflect an increasing dependence on protein degradation in order to support the high respiratory rates in later stages of fruit ripening. Furthermore, this difference in sugar accumulation suggests that the amount of sugars that accumulate in fruit is not only dependent on endogenous metabolic processes, but also affected by the degree of phloem unloading, since tomato fruit themselves possess a relatively low photosynthetic activity (Farrar and Williams, 1991; Lytovchenko *et al*., 2011).

Other notable differences between the differently ripened fruit were the lower levels of malate in red tomato fruit ripened on the vine. It has previously been reported that levels of malate, which makes an important contribution to taste, decrease during normal ripening (Carrari *et al*., 2006; Osorio *et al*., 2011). Moreover, putrescine levels were considerable higher at the B stage in off‐vine than in on‐vine ripening. Given its accumulation pattern in the ripening mutants described above, these results reinforce the potential importance of polyamines in ripening and further suggest that manipulating polyamine levels has considerable potential for fruit quality improvement under a range of cultivation practices.

### Long‐term prospects for improved tomato fruit quality

A major target of tomato breeding programmes has been yield and long postharvest shelf life, which has had unintended negative consequences for flavour and nutrient content (Emery and Munger, 1970). This deterioration of quality relates directly to complex genetic traits, and a current challenge for modern breeding programs is to deliver commercial varieties with improved flavour, but that maintain the high yield and extended shelf life (Klee and Tieman, 2018). Nowadays, due to the reduced cost of DNA sequencing, genome‐wide association studies (GWAS) have been made possible, in which superior alleles of genes controlling accumulation of key flavour metabolites in tomato fruits can be identified. Tieman *et al*. (2017), using heirloom and modern varieties including wild *S. lycopersicum* var. *cerasiforme,* and the most closely related species, *S. pimpinellifolium*, identified genetic loci that affect many of the most important acids, sugars and volatiles.

The well‐known *rin* and *nor* mutations are of horticultural interest as they block the ripening process, and cultivars harbouring these mutations yield fruits with slowed ripening and extended shelf life. These mutations have been bred into many commercial tomato varieties, although it is well established that they have undesirable effects on taste and flavour (Garg *et al*., 2008; Kovács *et al*., 2009). Here, we characterized the *dfd* mutant that exhibits ostensibly normal ripening, but little postharvest deterioration over many weeks. We show that in contrast to *nor* and *rin*, the *dfd* mutation results in extended shelf life without compromising sugar, acid and nutrient content. This suggests that there are opportunities for identifying new mutations, including novel alleles of well‐known mutations, for targeted improvement of one or multiple traits. Environmental variables can also play an important modulating effect on traits such as shelf life, although such phenomena are typically not well understood at the molecular level. For example, some studies have established that water availability during tomato cultivation influences shelf life; reporting that high water availability can lead to higher rates of fruit deterioration (Patané and Cosentino, 2010; Patané *et al*., 2011). Moreover, Conesa *et al*. (2014) reported that tomato shelf life is influenced by the irrigation regime during cultivation and also has a defined genetic component. In this regard, the *dfd* mutant will be a valuable genetic resource to elucidate the genetic control of extended shelf life without a reduction in fruit quality. Indeed, the demonstration that off‐vine ripening has such significant consequences on the chemical composition of the fruit implies that the genetic approach will likely ultimately be more suitable for the production of varieties that have both a long shelf life and high nutritional quality.

## Experimental procedures

### Plant materials

Tomato fruit (*S. lycopersicum*) M82, *dfd* (*delayed fruit deterioration*), AC (Ailsa Craig) and isogenic lines of the *nor* (*non‐ripening*) and *rin* (*ripening inhibito*r) mutants (cv. AC, backcross parent) were grown in a greenhouse at 26ºC/20ºC (day/night) with a 12‐h photoperiod. To collect stages prior to ripening, fruit were tagged at anthesis and harvested at 39 DAP (green; G) and immediately frozen in N_2_ liquid. Fruits picked at 42 DAP (breaker; B) were left to ripen at room temperature (fruit from ripening mutants were collected at equivalent DAP). Fruits were collected at different time points; B1 (breaker + 1 week; w), B2 (breaker + 2w), B3 (breaker + 3w), B4 (breaker + 4w), B6 (breaker + 6w) and B12 (breaker + 12w). Pericarp tissue was collected from all fruits, immediately frozen in N_2_ liquid and stored at −80ºC until further analysis. Transcript and metabolome analyses were performed on the same material. All fruits were collected from four individual plants for each genotype, with each fruit being considered a biological replicate.

#### Material for the M82 on‐/off‐vine ripening analysis

Tomato fruits were grown in the greenhouse and staged as above. For M82 off‐vine stages, fruit were harvested at 39 DAP (G), placed in open jars for 12 hours and 10 ppm ethylene was applied for 24 hour at which point the fruit were removed from the jars. After the fruits were ripened at room temperature, they were collected at different time points; 42 DAP (breaker; B), 46 DAP (turning; Tu), 47 DAP (pink; Pi) and 49 DAP (red ripe; RR). For the M82 on‐vine experiments, the fruit were tagged at 7 DAP and harvested at the same time points as the M82 off‐vine samples.

### Textural analysis

Firmness measurements were made as described in Saladié *et al*. (2007). Briefly, intact tomato fruit were tested four times at equidistant points along the equatorial plane of the fruit with a 50‐mm‐wide P50 DIA compression plate controlled by a Stable Microsystems Texture Analyzer (TA‐XT2i; Stable Micro Systems) loading at 1 mm/S and compressed to a vertical displacement of 1 mm. Firmness was defined as the response force to a 0.05 N applied force.

### Water loss measurements

Seven detached fruits from each genotype were kept at room temperature. Water loss per unit fruit surface area was calculated after measuring the weight decrease over time and measuring fruit dimensions.

### Metabolome analysis

A total of 250 mg of pericarp tissue per biological replicate was used. Metabolite extraction and derivatization for GC‐MS were performed as previously described (Osorio *et al*., 2012). Metabolites were identified by comparison to database entries of authentic standards, and relative quantification was performed.

### Carotenoid analysis

Carotenoids were extracted and analysed as described by Alba *et al*. (2005).

### RNA extraction and quantification for qRT‐PCR

RNA extraction and qRT‐PCR determination were carried out as described by Zanor *et al*. (2009). Briefly, the integrity of the extracted RNA was checked by electrophoresis under denaturing conditions after treating the RNA with DNAseI (Roche). First‐strand cDNA synthesis was performed from 500 μg of RNA according to the supplier's protocol (Bio‐Rad). Values showed in this publication are normalized to the expression of the *18SIF* (*Solyc06 g054400*) and *RPL2* (*Solyc10 g006580*) genes. The sequences of the primers used in the expression analysis are listed in Table S4.

### Data analysis and statistics

Data normalization, heat maps and correlation analysis based on Pearson correlation were performed using R software (Gentleman, 1996).

## Conflicts of interest

The authors declare no conflict of interest.

## Author contributions

S.O., R.C., A.L., R.M., I.S. and J.V. were involved in experimental design, data acquisition and analysis. S.O., J.G., A.F. and J.R, contributed to data interpretation, as well as drafting the manuscript and all authors contributed to editing the manuscript. All authors have given final approval of the version to be published and have agreed to be accountable for all aspects of the work, ensuring that questions related to the accuracy or integrity of any part of the work are appropriately investigated and resolved.

## Supporting information


**Figure S1** Heatmap of metabolite‐metabolite correlations across M82 fruit ripened on or off the vine.
**Figure S2** Expression of *SAM decarboxylase (Solyc05 g010420*) and *spermidine synthase (Solyc05 g005710*) genes by quantitative real‐time PCR (qRT‐PCR) in M82, *dfd*,* nor,* and *rin* genotypes at the green (39 DAP) and breaker (B) ripening stages, or equivalent DAP in the ripening mutants.

**Table S1** Loading plots of metabolites for the first two principle components, PC1 and PC2.

**Table S2** Comparison of carotenoid levels in fruit at the green, G; breaker, B; breaker + 1 week, B1; breaker + 2 weeks, B2; breaker + 3 weeks, B3; breaker + 4 weeks, B4; breaker + 6 weeks, B6; breaker + 12 weeks, B12 stages from *nor, rin*, and *dfd* mutants, AC (Ailsa Craig) and M82 varieties.

**Table S3** Comparison of primary metabolite composition of fruit at the green, G; breaker, B; breaker + 1 week, B1; breaker + 2 weeks, B2; breaker + 3 weeks, B3; breaker + 4 weeks, B4; breaker + 6 weeks, B6; breaker + 12 weeks, B12 stages from *nor, rin*, and *dfd* mutants, AC (Ailsa Craig) and M82 varieties.

**Table S4** List of PCR primers.
