# Supplementary material for: Genetic and metabolic effects of ripening mutations and vine detachment on tomato fruit quality
Source: Plant Biotechnol J. 2019 Jun 11;18(1):106–18. doi: 10.1111/pbi.13176 (PMC6920187; doi:10.1111/pbi.13176)
Supplement: Supplementary file 1 — Figure S1 Heatmap of metabolite‐metabolite correlations across M82 fruit ripened on or off the vine. Figure S2 Expression of SAM decarboxylase (Solyc05 g010420) and spermidine synthase (Solyc05 g005710) genes by quantitative real‐time PCR (qRT‐PCR) in M82, dfd, nor, and rin genotypes at the green (39 DAP) and breaker (B) ripening stages, or equivalent DAP in the ripening mutants. [file PBI-18-106-s004.pdf]

Supporting Information

Genetic and Metabolic Effects of Ripening Mutations and Vine Detachment on Tomato Fruit Quality

Sonia Osorio, Raphael T. Carneiro, Anna Lytovchenko, Ryan McQuinn, Iben Sørensen, José G. Vallarino, James J. Giovannoni, Alisdair R. Fernie, Jocelyn K.C. Rose

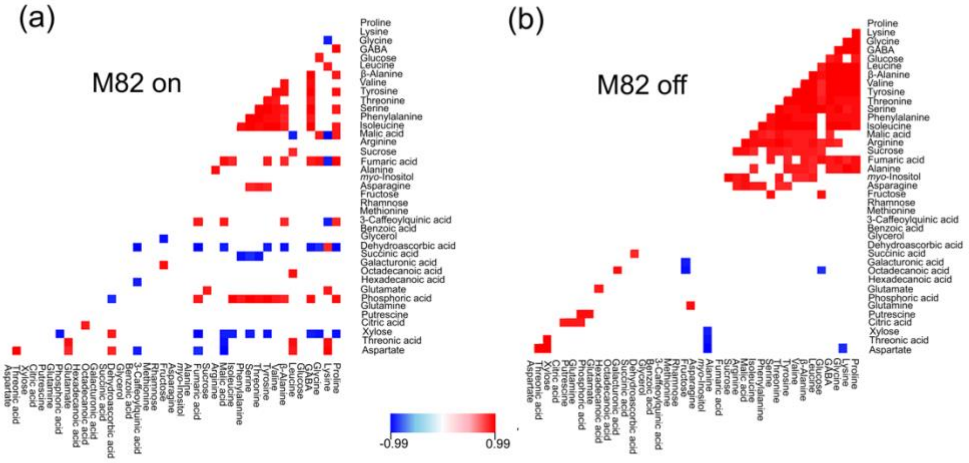

Figure S1. Heatmap of metabolite-metabolite correlations across M82 fruit ripened on or off the vine. Each square represents the correlation between the metabolite heading the column with the metabolite heading the row. Correlation coefficients and significance values were calculated by applying the Pearson algorithm using R. Only significant correlations are presented (P>0.05). Positive and negative correlations are presented in red and blue, respectively.

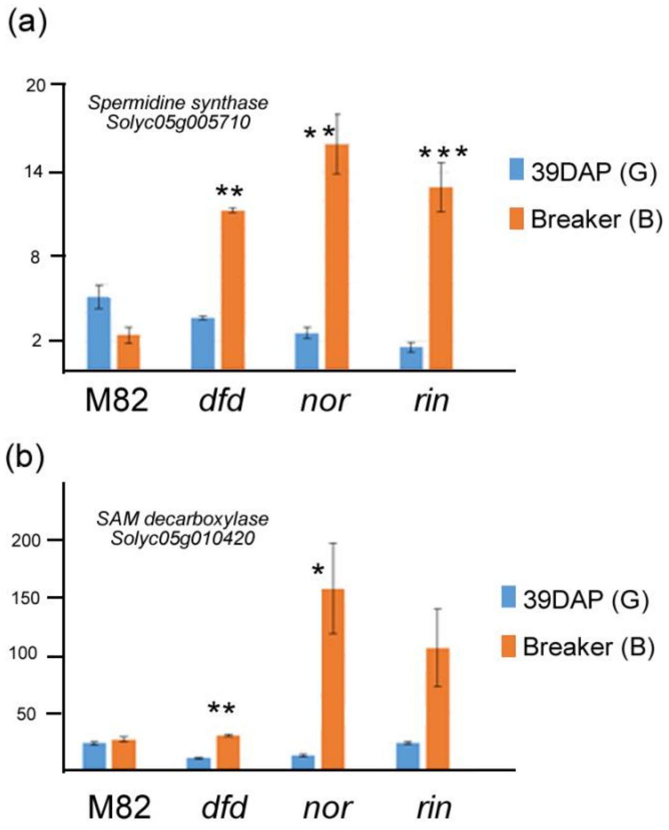

Figure S2. Expression of *SAM decarboxylase (Solyc05g010420*) and *spermidine synthase (Solyc05g005710*) genes by quantitative real-time PCR (qRT-PCR) in M82, *dfd*, *nor,* and *rin* genotypes at the green (39 DAP) and breaker (B) ripening stages, or DAP equivalent in the ripening mutants. Error bars indicate mean±SE of three biological replicates. The asterisks indicate significant differences by *t*-test analysis: (**P*<0.01; ***P*<0.05; ****P*<0.001).
